# A novel rabbit model of atherosclerotic vulnerable plaque established by cryofluid-induced endothelial injury

**DOI:** 10.1038/s41598-024-60287-0

**Published:** 2024-04-24

**Authors:** Huaizhi Lu, Yiran Xu, Hui Zhao, Xuesheng Xu

**Affiliations:** 1 Department of Cardiovascular Medicine, First People’s Hospital of Shangqiu, Kaixuan South Road 292, Shangqiu, 476000 China; 2 The Second Naval Hospital of Southern Theater Command of PLA, Sanya, 572029 China

**Keywords:** Vulnerable plaque, Animal model, Endothelial injury, Coronary heart disease, Acute coronary syndromes, Vascular diseases, Experimental models of disease

## Abstract

Acute thrombosis secondary to atherosclerotic plaque rupture is the main cause of acute cardiac and cerebral ischemia. An animal model of unstable atherosclerotic plaques is highly important for investigating the mechanism of plaque rupture and thrombosis. However, current animal models involve complex operations, are costly, and have plaque morphologies that are different from those of humans. We aimed to establish a simple animal model of vulnerable plaques similar to those of humans. Rabbits were randomly divided into three groups. Group A was given a normal formula diet for 13 weeks. Group C underwent surgery on the intima of the right carotid artery with – 80 °C cryofluid-induced injury after 1 week of a high-fat diet and further feeding a 12-week high-fat diet. Group B underwent the same procedure as Group C but without the – 80 °C cryofluid. Serum lipid levels were detected via ELISA. The plaque morphology, stability and degree of stenosis were evaluated through hematoxylin–eosin (HE) staining, Masson trichrome staining, Elastica van Gieson staining (EVG), and oil red O staining. Macrophages and inflammatory factors in the plaques were assessed via immunohistochemical analysis. The serum low-density lipoprotein cholesterol (LDL-C), triglyceride (TG), and total cholesterol (TC) levels in groups B and C were significantly greater than those in group A. No plaque formation was observed in group A. The plaques in group B were very small. In group C, obvious plaques were observed in the blood vessels, and the plaques exhibited a thin fibrous cap, a large lipid core, and partially visible neovascularization, which is consistent with the characteristics of vulnerable plaques. In the plaques of group C, a large number of macrophages were present, and matrix metalloproteinase 9 (MMP-9) and lectin-like oxidized LDL receptor 1 (LOX-1) were abundantly expressed. We successfully established a rabbit model of vulnerable carotid plaque similar to that of humans through the combination of cryofluid-induced endothelial injury and a high-fat diet, which is feasible and cost effective.

## Introduction

Acute cardiovascular and cerebrovascular events, recognized as among the leading causes of mortality worldwide, are closely linked to the genesis and progression of atherosclerosis^1,2^. With the expansion of research in this domain, it has been acknowledged by professionals that the primary risk factor for these acute events is not determined by the plaque's size but by its stability. The cause behind acute cardiac and cerebral ischemia is often acute thrombosis following plaque rupture^3–5^. In 1989, Muller et al. introduced the term 'vulnerable plaque' to denote a type of atherosclerotic plaque that is non-obstructive yet prone to rupture, typically featuring a large lipid core, a thin fibrous cap, and macrophage infiltration^6^. This concept has gradually gained acceptance in the academic community. Further clinical investigations have identified various pathomorphological types of plaques that could lead to acute coronary syndrome. Naghavi et al.^7^ later refined the 'vulnerable plaque' concept, describing plaques that are predisposed to thrombosis and have a high potential to rapidly evolve into the primary cause of acute cardio-cerebrovascular thromboembolic events. These vulnerable plaques, distinguished by their immunohistological and pathological characteristics, are categorized into several types, including^3,8^ parenchymatous atherosclerotic plaque, plaques with a large lipid core, plaque heavily infiltrated by inflammatory cells, neovascularised plaque, intra-plaque hemorrhage, and plaques with elevated inflammatory markers. While stable angina is marked by smooth fibrous coronary plaques, irregular or ruptured plaques are commonly associated with unstable angina^9^, and erosive plaques are often found in young sudden death victims^10^. Thus, the biological mechanism underlying plaque rupture is influenced by its histological morphology, including the thickness of the fibrous cap^11,12^, the properties and thickness of lipid core^13^, the properties of vascular wall tissue^14^, and the morphology of lesions.

The direct assessment of plaque heterogeneity in humans is challenging due to uncontrollable experimental variables and the slow disease progression, which may limit many studies. Consequently, reliable animal models are vital for advancing the understanding of plaque diagnosis and treatment^15^. Presently, established models for studying atherosclerotic plaque include the use of the classic aortic balloon strain with a high-fat diet^16^, a high-fat diet alone^17^, liquid nitrogen hypothermia damage with a high-fat diet^18^, and gene knockout mouse models^19,20^. These models face issues such as operational complexities, risks to researchers^18,21^, differences in plaque morphology compared to humans^21,22^, and high costs^23^, as elaborated in the Supplementary Material. Therefore, the development of an animal model that is both simple to implement and closely mirrors human plaque formation is urgently needed.

The endothelial injury response theory is widely acknowledged in the atherosclerosis mechanism^24^, suggesting that all major disease risk factors eventually lead to arterial intima damage, with atherosclerotic lesion formation being a response of the arteries to this damage. Fang SM established a human-like plaque model using liquid nitrogen to directly damage the vascular endothelium, combined with a high-fat diet, resulting in similar structure, cell composition, growth characteristics, and lipid accumulation pattern^18^. Trusal LR observed the damage of low temperature to the main organelles of endothelial cells through electron microscopy^25^. Hodl S found that low temperature can cause direct freezing damage and cell death through the formation of intracellular and extracellular ice crystals^26^. These studies have verified that low temperatures can induce endothelial injury. Based on this information, – 80 °C ethanol was employed as a cryofluid to damage rabbit carotid artery endothelium, in conjunction with a high-fat diet, to promote atherosclerotic plaque formation and development, aiming to establish a carotid atherosclerotic vulnerable plaque model.

## Materials and methods

### Preparation of animals

A total of 45 healthy male purebred New Zealand White rabbits weighing 3–4 kg were acquired from Zhengzhou Tuerkang Animal Husbandry Co., Ltd., and housed in the animal experimental center of Zhengzhou University under standard conditions. All of the protocols for testing met the relevant requirements of the Arterial Experimental Center of Zhengzhou University and were approved by the Animal Research Committee of Zhengzhou University (No. ZZU-LAC20220630[01]). All the experiments were performed in accordance with the relevant guidelines and regulations.

### Experimental design

According to a simple randomization method, the animals were divided into 3 groups of 15 animals each. Group A, the control group, was continuously fed a specific pathogen-free (SPF) rabbit maintenance formula diet for 13 weeks. Group B, the sham operation group, was subjected to carotid artery exposure surgery without intimal injury after 1 week of feeding on a high-fat diet (1% cholesterol, 8% lard, 10% egg yolk powder, and 81% SPF rabbit maintenance formula). After surgery, the rats were fed a high-fat diet for 12 weeks. In Group C, the cryofluid-induced injury group, which was fed a high-fat diet for 1 week, the right carotid artery intima was injured using – 80 °C ethanol. Group B, which underwent similar invasive surgery as Group C, did not experience intimal injury. Following surgery, they also continued on a high-fat diet for 12 weeks.

**Figure 1:**
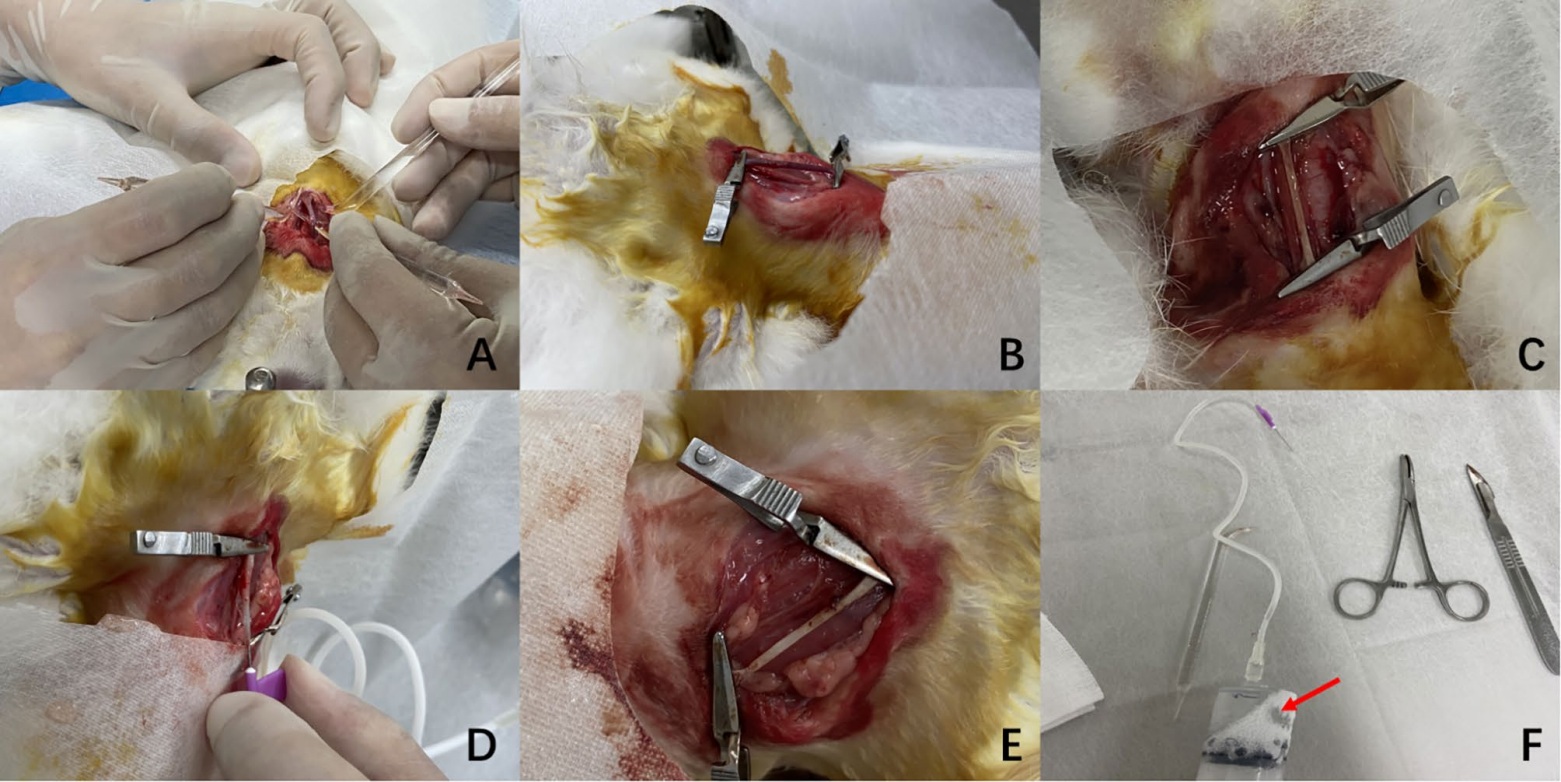
Procedures used to injure carotid artery endothelium. (**A**) Isolation of the carotid artery. (**B**) Both sides of the artery were clamped. (**C**) Vascular morphology after flushing the blood vessels. (**D**) A 50 mL syringe and infusion needle were used to fill the lumen with low-temperature ethanol. (**E**) Vascular morphology after hypothermic injury. (**F**) Main instrument. The syringe indicated by the arrow is filled with – 80 °C ethanol.

Prior to the procedure, the test animals were subjected to a fasting period of 12 h, while their access to water remained unrestricted. Anesthesia was induced by injecting 3% pentobarbital sodium (30 mg/kg) into the ear vein, followed by the isolation of approximately 4 cm of the right common carotid artery (Fig. 1A). To halt blood flow, clamps were first applied proximally to the heart and subsequently distally (Fig. 1B). Using the needle of a 1 mL syringe, a puncture was made at the distal end of the isolated artery, parallel to the vessel’s longitudinal axis. Blood was then withdrawn using the syringe, and the lumen was flushed with normal saline before being evacuated with the syringe (Fig. 1C). A needle for intravenous infusion was inserted into the vessel from a proximal position, parallel to its longitudinal axis, and left in place. Utmost care was taken during these procedures to prevent damage to the vessel. In Group C, – 80 °C ethanol was administered through the indwelling intravenous infusion needle until the carotid artery was saturated with the cryogenic liquid, which then exited through the distal puncture site (Fig. 1D). This entire process lasted for 4 min (Fig. 1E). Group B was not subjected to cryogenic injury.

Following the procedure, the artery’s lumen was flushed with normal saline again. The distal clamp was released first, and the puncture site was compressed with moist gauze for 3 min to control bleeding. If no bleeding was noted, the proximal clamp was then released, and the puncture site was similarly compressed to halt bleeding. After checking for occasional bleeding and confirming intra-arterial blood flow, the incision site was rinsed with penicillin saline, the wound was sutured, and disinfected with iodophor. Post-operatively, 400,000 units of penicillin sodium were administered intramuscularly for three days to prevent infection, and the high-fat diet regimen was maintained. The primary equipment used in the experiment is depicted in the Figure, including a 50 mL syringe connected to an infusion needle, which served as the low-temperature liquid dispensing device (Fig. 1F).

### Lipid levels

Blood samples were drawn from the middle auricular artery at two time points: at the initiation of the high-fat diet and 13 weeks later. The lipid levels in these samples were analyzed for each group utilizing reagents sourced from Shenzhen Redu Life Technology.

### Histopathological analysis

We anesthetized the rabbits by intravenous injection of pentobarbital and then sacrificed them by giving intravenous air. After the animals were sacrificed, paraffin-embedded and frozen sections were taken for HE staining (4 µm), Masson trichrome staining (4 µm), EVG staining (4 µm), and oil red O staining (8 µm). The H&E staining kit, Masson dye solution set, EVG dye set and oil red O dye set were obtained from Wuhan Servicebio Technology Co., Ltd. Image-Pro Plus 6.0 was used to calculate the percentage of plaque area and stenosis rate (%) = pixel area of plaque/pixel area of lumen × 100.

### Immunohistochemistry

Monoclonal antibodies against LOX-1 (Beijing Biosen Biotechnology Co., Ltd.), CD68 (Abcam Co., Ltd.) and MMP-9 (Beijing Biosen Biotechnology Co., Ltd.) were utilized for immunohistochemical staining of blood vessels from the test animals. The expression levels of CD68, MMP-9, and LOX-1 in the plaques were quantitatively analyzed. The immunohistochemical staining process is detailed in the Supplementary Material.

### Scanning and transmission electron microscopy

Certain sections were preserved in a 3% glutaraldehyde solution for the preparation of ultra-thin tissue sections, and the ultrastructure of the plaque site (70 nm) was examined using transmission electron microscopy. Additional details on section staining are provided in the Supplementary Materials.

### Statistical analysis

Continuous variables were reported as mean ± standard deviation (SD) or median (interquartile range [IQR]) and were analyzed using the grouped *t*-test or Wilcoxon rank-sum test. A P value of < 0.05 was deemed to indicate statistical significance. All analyses were conducted utilizing SPSS version 25.0 (IBM, Armonk, New York, USA).

### Ethical approval

The study is reported in accordance with ARRIVE guidelines.

## Results

### General information

Overall, the rabbits exhibited good health. Within Group C, one NZW rabbit died from overdose of anesthesia, and another to diarrhea. In Group B, diarrhea was the cause of death for only one rabbit. All other animals successfully completed the experiment, and data from 42 animals were analyzed (15 rabbits in Group A, 14 rabbits in Group B, and 13 rabbits in Group C). The average duration of the procedure for our animal model was approximately 25 min. The primary reagent used in the modeling process was – 80 °C ethanol, which served as the medium, and the associated costs were exceptionally low. Plaque formation was observed in all animals within Group C.

### Blood lipids

**Figure 2:**
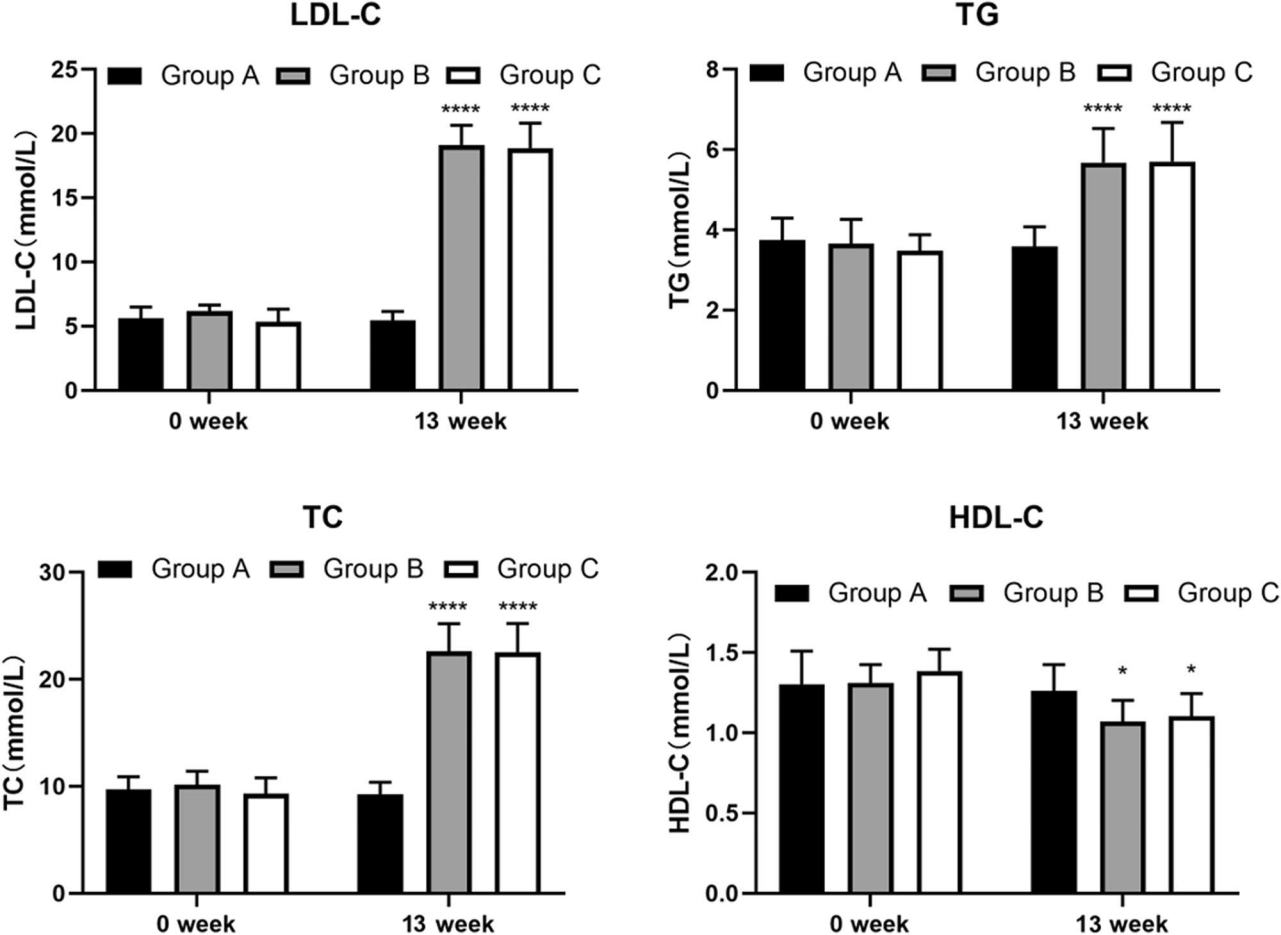
Blood lipid level in three groups. Data are presented as mean ± SEM. (*) means treated groups versus control group (Group A), Statistical significance was indicated by *P < 0.05, ***P < 0.001.

After 13 weeks, LDL-C, TG, and TC were significantly greater in Groups B and C than in Group A, but there was no significant difference between the two groups. High-density lipoprotein cholesterol (HDL-C) levels were lower in Group A than in Group A, but there was also no significant difference between the two groups (Fig. 2).

### Overview observation

The initial observational results indicated that in the control group, the right carotid artery wall appeared smooth, soft, and elastic. In the sham operation group, the right common carotid artery was largely unchanged, albeit with the presence of minor lipid streaks. In the group subjected to cryofluid-induced injury, there was a significant thickening and stiffening of the right common carotid artery wall, accompanied by an extensive presence of yellowish-white lipid-like material on the intimal surface. This material projected into the lumen and coalesced, forming characteristic atherosclerotic plaques. Additionally, small hemorrhagic spots were identified on the surface of some plaques, with small thrombi attached to them.

### Pathomorphological observation

**Figure 3:**
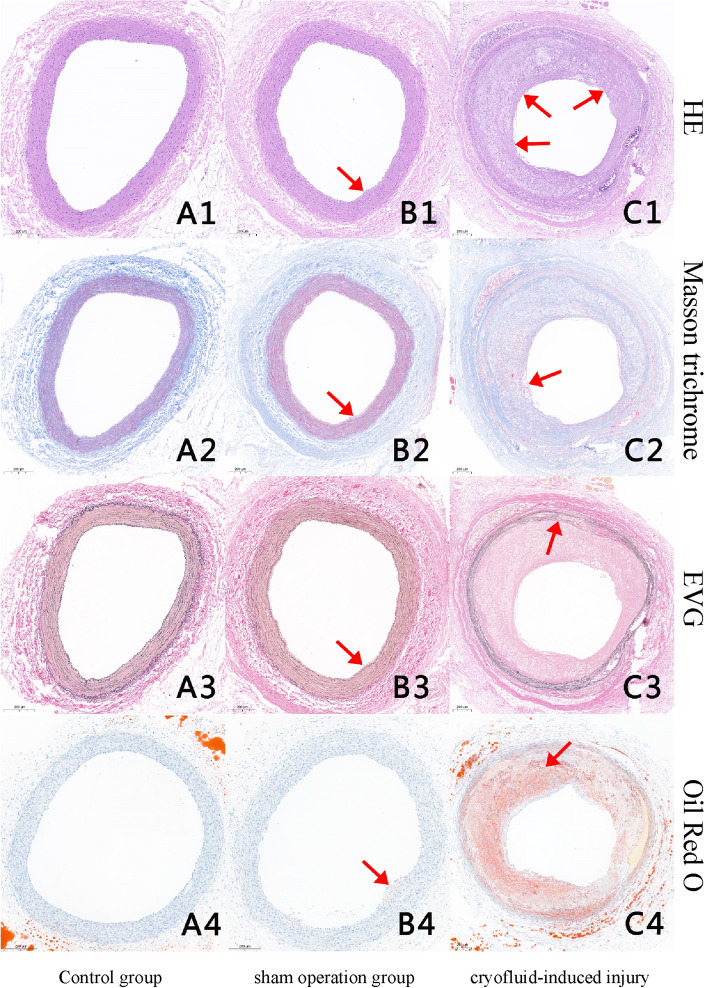
Four types of staining in carotid artery sections from the three groups (**A1**–**C1**) HE staining (magnification 40 ×): in the control group, the intima, media, and adventitia were intact, and no plaque formation was observed; in the sham operation group, endovascular plaques were formed, but the volume was small (arrow); in the cryofluid-induced injury group, evident plaque formation in the lumen (arrow). (**A2**–**C2**) Masson staining (magnification 40 ×): in the control group, the vascular wall was rich in collagen fibers; in the sham operation group, the plaques had more collagen fibers and thicker fibrous caps; in the cryofluid-induced injury group, thin fibrous cap with overall low content of collagen fibers in plaques (arrow). (**A3**–**C3**) EVG staining (magnification 40 ×): in the control group, the structure of the vascular elastic plate was complete; in the the ham operation group, elastic plates were relatively intact, with slight morphological changes; in the cryofluid-induced injury group, elastic plates damaged with the disappearance of city wall-like forms (arrow). (**A4**–**C4**) Oil red O staining (magnification 40 ×): in the control group, there was no lipid deposition in the blood vessels; in the sham operation group, the arrow points to a small lipid-stage plaque; in the cryofluid-induced injury group, plants rich in lipids that form a lipid core (arrow).

Group A: HE staining revealed complete and orderly arranged endothelial cells, a clear and intact inner elastic membrane, well-defined intermediate smooth muscle cells of regular morphology, and the absence of plaque formation or lipid deposition (Fig. 3A1–A4).

Group B: Endothelial hyperplasia was observed, along with small plaques in some vessels (Fig. 3B1), and subendothelial infiltration by circular foam cells. Masson’s trichrome staining highlighted abundant blue-stained collagen fibers in small plaques and a regular, hierarchical arrangement of medial smooth muscle cells and elastic fibers (Fig. 3B2). EVG staining did not show damaged elastic plates but revealed regular bending of intermediate elastic fibers without signs of vascular remodeling (e.g. stretching and straightening) and minimal lipid deposition (Fig. 3B3). These changes were consistent with the fatty streak stage and primary plaque formation (Fig. 3B4).

**Figure 4:**
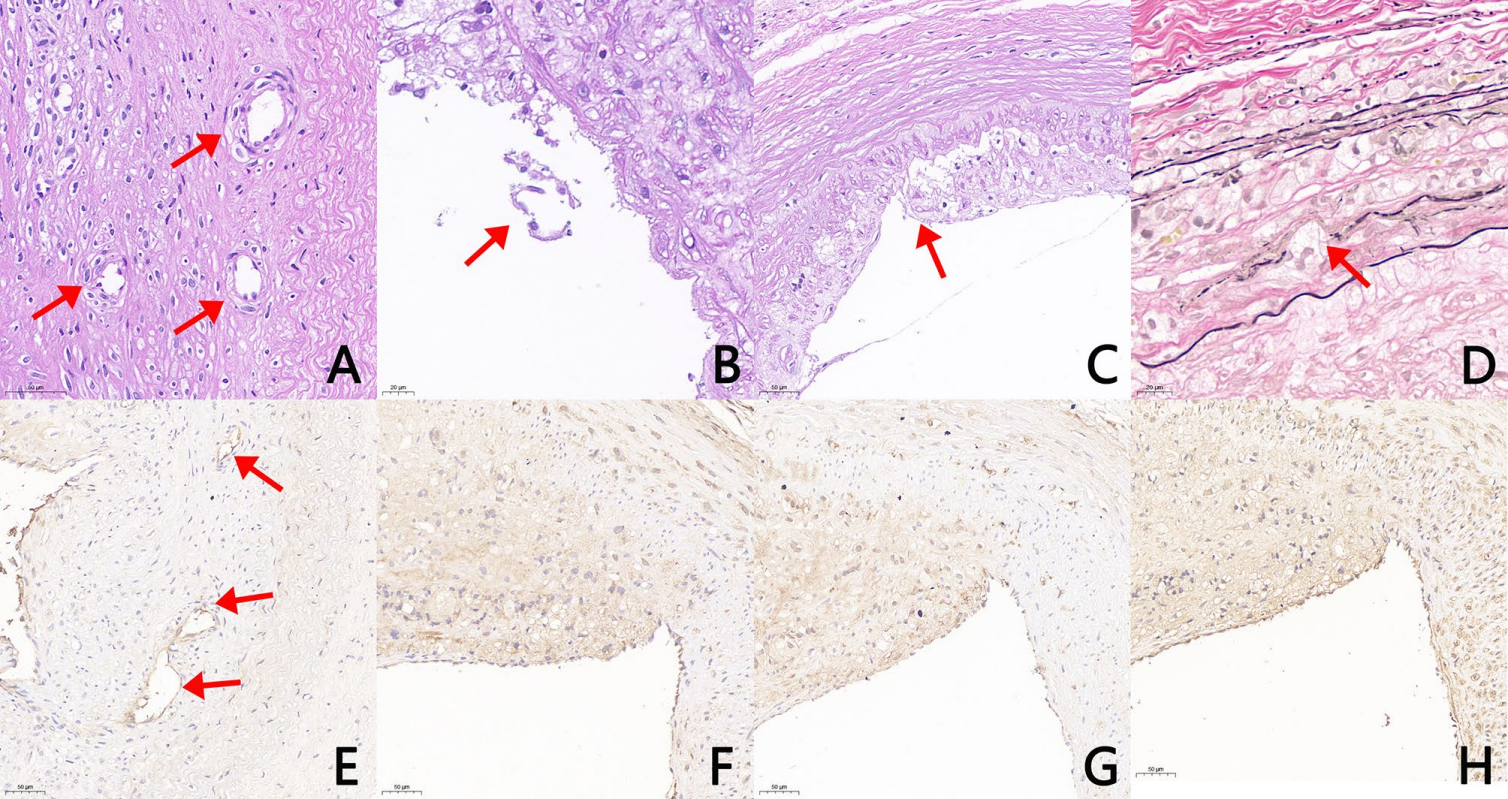
Images of the cryofluid-induced injury group with HE staining, EVG staining and Immunohistochemistry staining. HE staining: (**A**) Intraplaque neovascularization (arrow) (magnification 400 ×). (**B**) Endothelial cell shedding (arrow) (magnification 400 ×). (**C**) Rupture of the plaque shoulder (magnification 100 ×). (**D**) EVG staining: eroded and damaged internal elastic plate (magnification 400 ×). Immunohistochemistry staining: (**E**) CD31-labeled neovascularization within the plaque (arrow) (magnification 200 ×). (**F**) CD68-labeled macrophages (magnification 200 ×). (**G**) MMP-9 is abundantly expressed and colocalized with CD68 (magnification 200 ×). (**H**) LOX-1 is abundant in plaques (magnification 200 ×).

**Figure 5:**
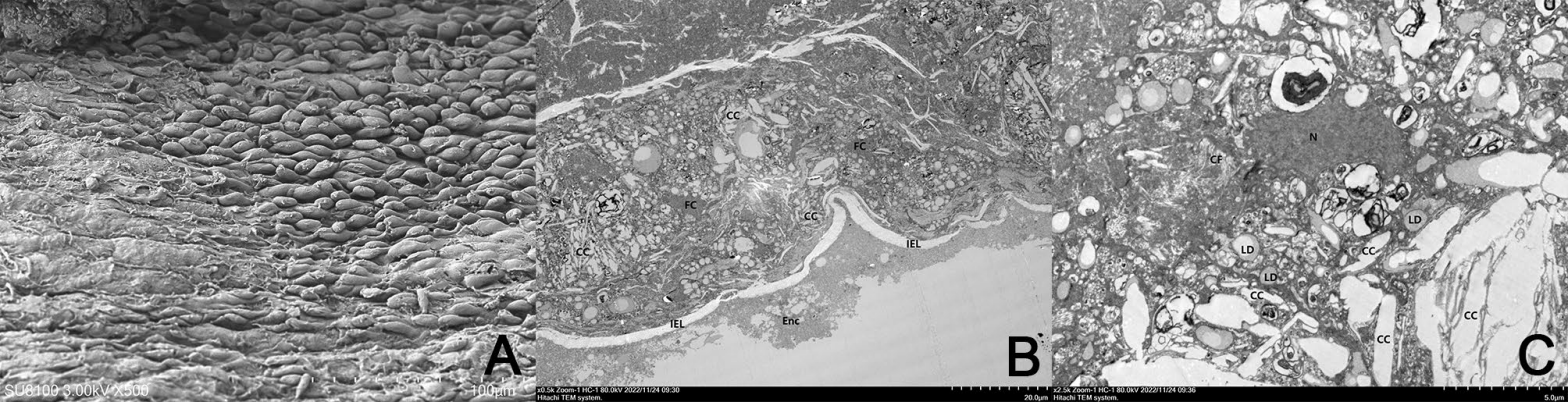
Scanning electron microscopy (SEM) and transmission electron microscopy (TEM) images of blood vessels in the cryofluid-induced injury group. (**A**) SEM image showing partial loss of endothelial cells. (**B**) TEM image: The intravascular elastic lamina (IEL) is disrupted, and macrophage-derived foam cells (FCs) and cholesterol crystals can be observed in the plaque (CC). (**C**) TEM: Plaques are rich in lipids (LDs) and contain cholesterol crystals (CCs).

In Group C, HE staining revealed typical unstable plaques manifested as thin fibrous caps and lipid cores (Fig. 3C1,C4). The intima was infiltrated by macrophages and various sources of foam cells with local mucoid degeneration and local small fissures in some patches (Figs. 3C1, 4F). Masson trichrome staining of the fiber cap revealed that the number of smooth muscle cells was significantly reduced, and some of the cells had scattered blue-dyed collagen fibers, some of which exhibited flaky mucus aggregation (Fig. 3C2). The results of EVG staining revealed a lamellar arrangement of elastic fibers and smooth muscle cells, with some damage to the inner elastic plates. We also observed that smooth muscle cells migrated into the inner membrane via the damaged elastic plates and exhibited clear blue‒black nuclei (Fig. 3C3). Some elastic fibers of the vascular media were pulled straight with thinner vascular media, showing vascular remodeling (Fig. 4D). The media even disappeared so that the intima met the adventitia. Neovascularization was also found in the plaques (Fig. 4A,E). Partial endothelial cell shedding was observed on the surface of the plaque (Figs. 4B, 5A), and shoulder rupture was observed in some plaques (Fig. 4C). CD68-labeled macrophage staining and transmission electron microscopic analyses helped us identify numerous macrophages in the plaque (Figs. 4F, 5B) and MMP-9 (Fig. 4G). There was a common area for distribution of the two. Moreover, LOX-1 was abundantly expressed in plaques (Fig. 4H).

The experimental outcomes did not demonstrate any notable differences in the lumen area between Group B and Group C. However, the plaque area in Group C was significantly larger, exhibiting an average stenosis percentage of 72.81%, in stark contrast to just 33.00% in Group B. This substantial disparity underscores the statistical significance of these findings in our analysis (Table 1).

**Table 1:** Plaque area and lumen area stenosis percentage of Groups B and C.

	Group A (mean ± SD)	Group B (mean ± SD)	Group C (mean ± SD)	Mean difference between groups (95% CI)	*P* value
Plaque area (pixel × 1000)	0.31 ± 0.13	60.53 ± 30.70	187.67 ± 34.38	127.14	< 0.0001
Lumen area (pixel × 1000)	237.73 ± 15.76	234.63 ± 85.67	263.24 ± 66.24	28.61	> 0.05
Stenosis percentage	0.13 ± 0.06	26.41 ± 13.07	72.81 ± 14.97	46.40	< 0.0001

*CI* confidence interval, *SD* standard deviation.

### Quantitative analysis of immunohistochemical plaques

In the quantitative analysis of immunohistochemical plaques, the primary evaluation metrics included two aspects: the positive cell ratio, calculated as the number of positive cells divided by the total number of cells, and the positive cell density, determined as the number of positive cells per area of tissue tested. The ratio and density of LOX-1, MMP-9, and CD68 positive cells in Group C were significantly higher than those observed in Group B (Table 2).

**Table 2:** Immunohistochemical quantitative analysis of LOX-1, MMP-9, and CD68 (Positive Cells Density, number/mm^2^).

		Group A	Group B	Group C	t value	*P* value
LOX-1	Positive cells, %	3.2 ± 0.8%	34.8 ± 4.8%	87.2 ± 9.6%	– 9.778	< 0.0001
	Positive cells density	48.8 ± 10.4	467.8 ± 187.1	1393.3 ± 298.6	− 5.254	0.002
MMP-9	Positive cells, %	2.3 ± 0.9%	26.1 ± 7.6%	87.1 ± 6.8%	− 12.013	< 0.0001
	Positive cells density	43.6 ± 7.4	453.3 ± 121.5	1185.0 ± 334.6	− 3.667	0.010
CD68	Positive cells, %	1.5 ± 0.23%	19.4 ± 12.3%	89.2 ± 10.4%	− 7.398	< 0.0001
	Positive cells density	28.0 ± 3.6	214.0 ± 100.8	812.0 ± 179.8	− 5.803	0.001

### Scanning and transmission electron microscopy

The intima was severely damaged, with endothelial cells (ENCs) mostly necrotic and disintegrated (Fig. 5A), a widened intercellular space and a loss of connections. The internal elastic membrane (IEL) was locally fractured and discontinuous (Fig. 5B); a large number of foam cells (FC) gathered in the media, as did a large number of intracellular cholesterol crystals (CCs) and lipid droplets (LDs) (Fig. 5B,C).

## Discussion

Our study introduced an innovative rabbit carotid atherosclerotic plaque model through the combination of ethanol-induced vascular endothelium injury at – 80 °C coupled with a high-fat diet. It has been reported that the formation of atherosclerosis is affected by many factors, and hyperlipidemia is currently recognized as an important clinical factor^27–29^. Therefore, hyperlipidemia is an important prerequisite for the establishment of an atherosclerotic plaque model. Endothelial injury is also currently recognized as a factor that triggers atherosclerosis. In this study, we first used a high-fat diet to increase LDL-C levels in rabbits to establish atherosclerotic plaques, while low-temperature liquid was used to damage the vascular endothelium. To create a rabbit arteriosclerosis-vulnerable plaque model.

After 13 weeks on a high-fat diet, the levels of LDL-C, TC, and TG in rabbits from both the sham operation group and the cryogenic fluid injury group were significantly higher than those in the normal diet group. No significant difference was observed between the sham operation group and the cryogenic fluid injury group, suggesting that the surgical procedure did not influence the alterations in blood lipid levels. This indicates a high sensitivity of rabbits to high-fat diets, aligning with findings from other studies^30,31^. Consequently, many models for atherosclerotic plaque incorporate hyperlipidemia as a foundational aspect, employing various methods like the classic balloon strain method and liquid nitrogen injury method, with minor variations in high-fat feed recipes^18,32^.

The atherosclerotic plaques developed in this study bear the hallmarks of vulnerable plaques, including a thin fibrous cap, a large lipid core, macrophage infiltration, neovascularization, and the formation of cholesterol crystals^33,34^. In the model group, prominent atherosclerotic plaque formation and significant vascular stenosis were observed in the rabbit carotid arteries. HE staining revealed that the plaque’s fibrous cap was relatively thin, comprising a single layer of endothelial cells and a few smooth muscle cells. Masson-trichrome staining indicated a deficiency in collagen fibers in the fibrous cap, particularly at its thinnest point at the junction between normal vascular wall tissue and plaque or at the plaque's corner. As corroborated by previous research, the dynamic forces of cardiac systole and diastole, along with the shear force of coronary blood flow, may lead to fibrous cap rupture due to compression changes^35,36^. Oil Red O staining and transmission electron microscopy revealed substantial lipid deposition within the plaque, characterized by a large LDL-rich lipid core, abundant foam cells in sticky atheromatous substances, necrotic tissues, cholesterol crystals, and degraded blood components. The presence of a large lipid core, indicative of plaques vulnerable to rupture and potential thrombosis, was noted alongside severe myxoid degeneration, rendering the plaques unstable^37^. HE staining and scanning electron microscopy identified shed endothelial cells, surface layer erosion, and dispersed fibrin aggregation with entangled red blood cells and platelets. Following endothelial damage, no significant morphological or structural changes were observed; however, the properties related to vasodilation, anticoagulation, anti-leukocyte and platelet adhesion, anti-inflammation, and anti-smooth muscle cell proliferation were altered, leading to coronary artery constriction due to various factors. These combined effects predisposed the plaques to rupture, triggering thrombosis^38^. Virmani et al. performed an autopsy in patients with acute coronary syndrome resulting from plaque erosion, characterized by a rich smooth muscle and proteoglycan matrix and either reduced or absent endothelium on eroded plaque surfaces; the results suggested the critical role of the endothelium in preventing vulnerable plaques^39^.

In addition, neovascularization within plaques is an important indicator of intraplaque hemorrhage and plaque instability. In our model group, neovascularization was further observed in the plaques. According to Nie et al. hypoxic macrophages deep in the necrotic core may secrete a hypoxia-inducible factor that can induce neovascularization^40^.Without the support of elastic layers and smooth muscle cells, the walls of new vessels became more permeable; thus, allowing blood cholesterol, cytokines, and white blood cells to enter the necrotic core directly. The cholesterol crystals formed by free cholesterol may disrupt biofilms, erode fibrous caps, and penetrate the lumen, leading to embolism or thrombosis^41^ and providing key elements for sustained plaque progression. This can destabilize the plaque and cause its rupture. OCT has shown that the incidence of rupture of plaques with neovascularization is significantly greater than that without neovascularization, and for plaques with neovascularization, the fibrous caps are thinner^42^. By transfecting VEGF-A, FGF-2 and PDGF-BB into rabbit carotid plaques via a viral vector, Moreno et al.^37^ found that the overexpression of VEGF-A and FGF-2 promoted neovascularization in plaques, leading to hemorrhage and rupture.

The accumulation of inflammatory cells and matrix metalloproteinases (MMPs) within plaques is a key factor contributing to plaque vulnerability. In our model group, HE staining, CD68-labeled macrophage immunohistochemistry, and transmission electron microscopy revealed a high concentration of macrophages in the plaques, indicating significant inflammation. Additionally, elevated expression of LOX-1, a scavenger receptor crucial for the uptake of oxidized low-density lipoprotein cholesterol (ox-LDL) by arterial cells, was detected within the plaques. There is growing evidence of LOX-1's essential role throughout the progression of atherosclerosis, from its initiation to the destabilization of plaques. Research into the genetic makeup of LOX-1 has unveiled various genetic polymorphisms that could influence the risk of atherosclerotic cardiovascular events^43,44^. Moreover, MMP-9 expression was notably high in the plaques we analyzed. The infiltration of inflammatory cells, particularly macrophages, can lead to the degradation of the extracellular matrix and weaken the fibrous cap through phagocytosis or the secretion of fibrinolytic activators and MMPs, characteristic of plaques prone to rupture^45–48^. In comparison to the sham operation and the blank control groups, our model group exhibited an increased number of vasa vasorum in the adventitia, potentially accelerating the development of atherosclerosis and offering a crucial pathway for inflammatory cells to infiltrate the plaque^49^.

In general, compared to other rabbit models of atherosclerotic vulnerable plaques, our model stands out various advantages such as being more practical, safe, straightforward, and cost-effective. For example, the balloon injury plus high-fat diet method, commonly employed to induce atherosclerosis in rabbits, offers a high success rate. However, the predictability of plaque formation with this technique is less reliable than with other methods. The tools required for this method, such as balloons and guidewires, are relatively delicate and demand considerable skill from the researcher^32^. Additionally, the variability in blood vessel diameters among experimental rabbits complicates the control of injury severity and increases the risk of damaging other structures, such as the intraarterial elastic plate and tunica media^50^. Another animal model, the liquid nitrogen frostbite plus high-fat diet method can successfully create a model of vulnerable atherosclerotic plaque^18,51^, but it is associated with higher mortality and stroke rates. Liquid nitrogen poses a chemical hazard, necessitating specialized equipment for its use and storage, as well as specific training for operators. In the modeling surgery, the quantity and exposure time of liquid nitrogen must be meticulously regulated to prevent excessive endothelial damage and vascular wall necrosis. Additionally, adherence to stringent operating procedures for liquid nitrogen is essential to avert various accidents, such as frostbite and inhalation. Methods involving air drying are more prone to induce vasculitis than atherosclerotic plaque formation^52^. The vascular stenosis resulting from such methods significantly differs from the development of stenosis in human blood vessels. Furthermore, the surgical operation is technically challenging, requiring researchers to develop relevant skills and experience^53^, thereby limiting its generalizability. In contrast, our animal model employed cryofluid-induced injury to the vascular endothelium, combined with a high-fat diet, to establish a model of vulnerable plaque. Utilizing – 80 °C ethanol as the medium, this method circumvents the high risks associated with liquid nitrogen. The damage it causes is controllable, leading to a reduced incidence of cerebral infarction and death, and enhancing the model's uniformity. The procedure is straightforward, enabling even novice researchers to complete the modeling work according to provided instructions. Compared to balloon injury, liquid nitrogen damage, and gene knockout mouse models, the cost of this modeling approach is significantly lower, making it suitable for widespread adoption. From the analysis presented, it is clear that the cryofluid-induced injury method combined with a high-fat diet developed in this study effectively induces the formation of plaques that resemble those found in humans. However, there are also many limitations, such as a high risk of infection which required strict aseptic procedures and damage to the surrounding tissues because of the leak of low-temperature ethanol. However, despite the above shortcomings, as a valuable platform for scientific investigation, this model offers potential for various studies on vulnerable plaques, including the evaluation of drugs' effects on plaque stability.

### Supplementary Information

Supplementary Information 1.

Supplementary Information 2.

Supplementary Information 3.

Supplementary Information 4.

Supplementary Information 5.

Supplementary Information 6.

Supplementary Information 7.

## Data Availability

The data that support the findings of this study are available from the corresponding author, [Huaizhi Lu], upon reasonable request.
