# Supplementary material for: A novel rabbit model of atherosclerotic vulnerable plaque established by cryofluid-induced endothelial injury
Source: Sci Rep. 2024 Apr 24;14:9447. doi: 10.1038/s41598-024-60287-0 (PMC11043414; doi:10.1038/s41598-024-60287-0)
Supplement: Supplementary file 1 — Supplementary Information 1. [file 41598_2024_60287_MOESM1_ESM.pdf]

Comparison of shortcomings of major modeling methods

| Modeling method | Specific operations | shortcoming |
|---|---|---|
| High-fat diet+liquid nitrogen damages vascular endothelium (rabbit) | Under high-fat diet conditions, liquid nitrogen was used to directly damage the vascular endothelium, and the high-fat diet continued for 8 weeks. | The liquid nitrogen injury step has certain risks for the operator. At the same time, the operation is poorly controllable and the animal mortality rate is high. |
| High-fat diet+balloon straining vascular endothelium (rabbit) | Keep feeding on a high-fat diet, perform aortic endothelial balloon strain surgery after 0-3 weeks, and continue feeding for -14 weeks after surgery | The operation is complicated and can easily cause death of animals. |
| High-fat diet+dissection and air-drying of right femoral artery endothelium (rabbit) | After maintaining a high-fat diet for 2-4 weeks, the right femoral artery was dissected and air-dried to damage the endothelial cells, and the animals were continued to be fed for 6 weeks after surgery. | During the process of endothelial drying, the fragility of blood vessels increases, easily causing arterial rupture. |
| High-fat diet+carotid artery fixation (rabbit) | A silicone rubber ring was placed around the left common carotid artery of the rabbit and fixed with silk thread to cause vascular stenosis. The rabbit was fed high-fat feed for 8 weeks after surgery. | Limited to the study of atherosclerotic plaques caused by hemodynamic changes. |
| high fat diet （pig） | 10% lard + 0.75% cholesterol | expensive |
| $ApoE^{-/-}$ mice | 5%-40% fat + 0.15%-4% cholesterol, sugars, proteins, etc. can be added. | The modeling cycle is long and is greatly affected by the feed formula. |
