# Supplementary material for: A novel rabbit model of atherosclerotic vulnerable plaque established by cryofluid-induced endothelial injury
Source: Sci Rep. 2024 Apr 24;14:9447. doi: 10.1038/s41598-024-60287-0 (PMC11043414; doi:10.1038/s41598-024-60287-0)
Supplement: Supplementary file 2 — Supplementary Information 2. [file 41598_2024_60287_MOESM2_ESM.pdf]

Paraffin section immunohistochemical experiment report

I Experimental equipments and reagents

1.1 Experimental equipments

Name	Manufacturer	Model
dehydrator	DIPATH, Italy	Donatello
Embedding machine	Wuhan Junjie Electronics Co., LTD	JB-P5
Pathological microtome	Shanghai Leica Instrument Co., LTD	RM2016
Frozen table	Wuhan Junjie Electronics Co., LTD	JB-L5
Tissue machine	Zhejiang Jinhua Kedi Instrument Equipment Co., LTD	KD-P
Oven	Shanghai Huitai Instrument Manufacturing Co., LTD	DHG-9140A
slide	Servicebio	G6012
Cover glass	Jiangsu Shitai experimental equipment Co., LTD	10212432C
Microwave oven	Galanz microwave electric appliance Co., LTD	P70D20TL-P4
Decolorizing table	Servicebio	DS-2S100
Vortex mixer	Servicebio	MV-100
Palm centrifuge	Servicebio	D1008E
Pipette gun	Servicebio	
Tissue pencil	Servicebio	G6100
Microscope	Nikon	E100

1.2 Main experimental reagents

Name	Manufacturer	Model
Anhydrous ethanol	SCRC	100092683
Environmentally friendly dewaxing transparent liquid	Servicebio	G1128

xylene	SCRC	10023418
n-butanol	SCRC	100052190
Hydrochloric acid	SCRC	10011028
20×Citric Acid Antigen Repair Solution (pH 6.0)	Servicebio	G1202
20×Tris-EDTA Antigen Repair Solution (pH 9.0)	Servicebio	G1203
20×Tris-EDTA Antigen Repair Solution (pH 8.0)	Servicebio	G1206
PBS buffer	Servicebio	G0002
Universal Tissue Fixative (Neutral)	Servicebio	G1101
Bovine serum albumin BSA	Servicebio	GC305010
Normal rabbit serum (concentrated)	Servicebio	G1209
Hematoxylin dye	Servicebio	G1004
Hematoxylin differentiation solution	Servicebio	G1039
Hematoxylin blue return solution	Servicebio	G1040
Super clean fast drying sealant	Servicebio	G1404-100mL
DAB chromogenic reagent for histochemical kit	Servicebio	G1212

1.3 Antibody information and repair conditions

Antigen repair condition	Name of primary antibody	First anti item number	Primary antibody manufacturer	A resistant species	The dilution ratio of one antibody	Name of the corresponding secondary antibody
EDTA (pH9.0) Microwave medium fire for 5 minutes, cease fire for 5 minutes, then turn to low fire for 10 minutes	CD31	bs-0195R	Bioss	rab	1:200	HRP-labeled goat anti-rabbit IgG
EDTA (pH9.0) Microwave medium fire for 5 minutes, cease fire for 5 minutes, then turn to low fire for 10 minutes	CD68	ab213363	abcam	rab	1:5000	HRP-labeled goat anti-rabbit IgG
Citric acid (pH 6.0) Microwave medium heat for 8 minutes, cease fire for 8	MMP-9	GB11132	Servicebio	rab	1:1000	HRP-labeled goat anti-rabbit IgG

minutes, turn to medium-low heat for 7 minutes						
EDTA (pH9.0) Microwave medium fire for 5 minutes, cease fire for 5 minutes, then turn to low fire for 10 minutes	LOX-1	bs-2044R	Bioss	rab	1:200	HRP-labeled goat anti-rabbit IgG

(Note: The order of adding antibody samples is in accordance with the writing order of antibodies, the number of the second antibody, manufacturer and dilution ratio are shown in the following table)

II Experimental procedure

2. 1 Paraffin sections dewaxing to water: Put the sections into environmentally friendly dewaxing solution I10min- Environmentally friendly dewaxing solution II10min- Environmentally friendly dewaxing solution III10min- anhydrous ethanol I5min- anhydrous ethanol II5min- anhydrous ethanol III5min- distilled water in turn.

2.2 Antigen repair: The repair is shown in the table above. During this process, the buffer should be prevented from excessive evaporation and should not be dried. After natural cooling, the slide was placed in PBS (PH7.4) and washed by shaking on the decolorizing shaker for 3 times, 5min each time. (Repair fluid and repair conditions are determined according to the tissue)

2.3 Blocking endogenous peroxidase: The slices were placed in 3% hydrogen peroxide solution, incubated at room temperature away from light for 25 min, and the slides were placed in PBS (PH7.4) and washed three times on a decolorizing shaking table for 5min each time.

2.4 Serum closure: The tissue was uniformly covered with 3%BSA in the tissue chemical circle and closed at room temperature for 30min. (Primary antibody of goat origin is blocked with rabbit serum, other sources are blocked with BSA)

2.5 Add primary antibody: Gently shake off the sealing solution, add PBS to the section in a certain proportion of primary antibody, and the section is placed flat in a wet box at 4°C for overnight incubation.

2.6 Adding secondary antibody: The slide was placed in PBS (PH7.4) and washed by shaking on the decolorizing shaker for 3 times, 5min each time. After the slices were slightly dried, the tissue was covered with the secondary antibody (HRP label) of the corresponding species of the primary antibody, and incubated at room temperature for 50min.

2.7 DAB color development: The slide was placed in PBS (PH7.4) and washed by shaking on the decolorizing table for 3 times, 5min each time. After the sections were slightly dried, the freshly prepared DAB color developing solution was added into the circle. The color developing time was controlled under the microscope. The positive color was brown and yellow, and the sections were rinsed with tap water to terminate the color development.

2.8 Restaining nuclei: hematoxylin restaining for about 3min, washing with tap water, hematoxylin differentiation solution for a few seconds, rinse with tap water, hematoxylin return to blue solution, and rinse with running water.

2.9 Dewatering and sealing: Put the slices into 75% alcohol for 5min--85% alcohol for 5min-- anhydrous ethanol for 5min-- anhydrous ethanol for 5min-- n-butanol for 5min-- xylene for 5min to dehydrate and transparent, take the slices out of xylene to dry slightly, and seal the slices with glue.
2. 10 Microscopy: The results are interpreted under a white light microscope.

III Interpretation of results

The nucleus of hematoxylin stain is blue, and the positive signal of DAB is brown-yellow.

Attached table

Second antibody and TSA name	Model	Manufacturer	Dilution ratio
CY3 Labeled Goat Anti-Rabbit IgG	GB21303	Servicebio	1:300
CY3 tagged Goat anti-mouse IgG	GB21301	Servicebio	1:300
CY3 Labeled Goat anti-rat IgG	GB21302	Servicebio	1:300
CY3 Labeled Donkey Anti-Goat IgG	GB21404	Servicebio	1:300
Cy3-labeled Donkey anti-mouse IgG	GB21401	Servicebio	1:300
CY3 Labeled Donkey Anti-Rabbit IgG	GB21403	Servicebio	1:300
Alexa Fluor 488 Labeled Goat Anti-Rabbit IgG	GB25303	Servicebio	1:400
Alexa Fluor 488 labeled Goat anti-mouse IgG	GB25301	Servicebio	1:400
CY5 Labeled Goat anti-mouse IgG	GB27301	Servicebio	1:400
CY5 Labeled Goat anti-Rabbit IgG	GB27303	Servicebio	1:400
HRP * Polyclonal Rabbit Anti-Goat IgG	GB23204	Servicebio	1:200
Goat anti-mouse IgG labeled by HRP	GB23301	Servicebio	1:200
HRP * Polyclonal Goat anti-Rat IgG	GB23302	Servicebio	1:200
HRP was used to label goat anti-rabbit secondary antibody	GB23303	Servicebio	1:200
HRP was used to detect donkey antibody against goat antibody	GB23404	Servicebio	1:200
FITC * Donkey Anti-Goat IgG	GB22404	Servicebio	1:200
Fitc-labeled Goat anti-rat IgG	GB22302	Servicebio	1:200
Fitc-labeled Donkey Anti-Rabbit IgG	GB22403	Servicebio	1:200
Fitc-labeled Donkey Anti-Mouse IgG	GB22401	Servicebio	1:200
Alexa Fluor 594 Labeled Goat Anti-Rabbit IgG	111-585-003	Jackson	1:400
Alexa Fluor 594 labeled Goat anti-mouse IgG	115-585-003	Jackson	1:400

CY3-Tyramide	G1223	Servicebio	1:500
iF488-Tyramide	G1231	Servicebio	1:500
iF647-Tyramide	G1232	Servicebio	1:400
FITC-Tyramide	G1222	Servicebio	1:500
