# Supplementary material for: A novel rabbit model of atherosclerotic vulnerable plaque established by cryofluid-induced endothelial injury
Source: Sci Rep. 2024 Apr 24;14:9447. doi: 10.1038/s41598-024-60287-0 (PMC11043414; doi:10.1038/s41598-024-60287-0)
Supplement: Supplementary file 3 — Supplementary Information 3. [file 41598_2024_60287_MOESM3_ESM.pdf]

EVG staining experiment report

1 Apparatus and reagents

1.1 Major apparatus

Name	Producer	Model
Dehydrator	DIAPATH	Donatello
Embedding machine	Wuhan Junjie Electronics Co., Ltd	JB-P5
Pathology slicer	Shanghai Leica Instrument Co., Ltd	RM2016
Frozen platform	Wuhan Junjie Electronics Co., Ltd	JB-L5
Tissue spreader	Zhejiang Kehua Instrument Co., Ltd	KD-P
Oven	Tianjin Laibo Rui Instrument Equipment Co., Ltd	GFL-230
Frozen microtome	Thermo Fisher Technology (China) Co., LTD	CRYOSTAR NX50
Adhesive slide (paraffin section) (white paint band)	Servicebio	G6012-1
Adhesive slide (frozen section) (white paint ribbon)	Servicebio	G6012-2
Cover glass	Jiangsu Shitai experimental equipment Co., LTD	10212432C
Upright optical microscope	Nikon	NIKON ECLIPSE E100
Imaging system	Nikon	NIKON DS-U3

1.2 Major reagents

Name	Producer	Code
Ethanol	SCRC	100092683
Xylene	SCRC	10023418
Environmental Friendly Dewaxing Transparent Liquid	Servicebio	G1128-1L
Universal tissue fixative	Servicebio	G1101
EVG dye set	Servicebio	G1042
Neutral gum	SCRC	10004160

2. Preparation of tissue sections

The corresponding tissue sections were prepared according to the experimental SOP of Xavier's pathological tissue, such as fixation, embedding, paraffin section, frozen section, etc.

3. dyeing steps

3. 1 paraffin sections dewaxing to water: Put the sections into Environmental Friendly Dewaxing Transparent Liquid I 20min - Environmental Friendly Dewaxing Transparent Liquid II 20min - anhydrous ethanol I 5min - anhydrous ethanol II 5min - 75% alcohol for 5min, wash with tap water.

Rewarming and fixing frozen sections: the frozen sections were removed from the -20° refrigerator and restored to room temperature, fixed with tissue fixating solution for 15min, and then rinsed with running water.

3.2 EVG dyeing: EVG dye solution A: EVG dye solution B: EVG dye solution C5:2:2 Mixed into EVG dye solution (configured two days in advance), slice into EVG dye solution for 5min, rinse with tap water.

3.3 background differentiation: EVG dye B diluted twice after slightly differentiation, wash with tap water, so repeated operation, under the microscope to control the degree of differentiation, until the elastic fiber is purplish black, the background is gray and white nearly colorless.

3.4 Re-dyeing VG: Add EVG dye E 9ml and add EVG dye D 1ml to form VG dye (according to the proportion of the amount used), dyeing 1-3min (dyeing time depends on the composition of elastic fiber in the tissue, dyeing time is too short collagen color is light, dyeing time is too long elastic fiber will fade), rapid water washing, Three tanks of anhydrous ethanol for rapid dehydration.

3.5 transparent seal: two cylinders of clean xylene transparent 20s, 5min (xylene exclusive not shared with other xylene), neutral gum wet seal.

3.6 Microscopy, image acquisition and analysis.

4. Interpretation of results:

The elastic fibers are purplish black, the collagen fibers are red, and the background is yellow.

5. Precautions:

When differentiating, the elastic fiber is purplish black fine filmy, can not be over-differentiated, the elastic fiber fades, if the differentiation is insufficient, the VG redyeing effect is not good.
