# Supplementary material for: A novel rabbit model of atherosclerotic vulnerable plaque established by cryofluid-induced endothelial injury
Source: Sci Rep. 2024 Apr 24;14:9447. doi: 10.1038/s41598-024-60287-0 (PMC11043414; doi:10.1038/s41598-024-60287-0)
Supplement: Supplementary file 4 — Supplementary Information 4. [file 41598_2024_60287_MOESM4_ESM.pdf]

HE staining experiment report

1. Apparatus and reagents

1.1 Major apparatus

Name	Producer	Model
Dehydrator	DIAPATH	Donatello
Embedding machine	Wuhan Junjie Electronics Co., Ltd	JB-P5
Pathology slicer	Shanghai Leica Instrument Co., Ltd	RM2016
Frozen platform	Wuhan Junjie Electronics Co., Ltd	JB-L5
Tissue spreader	Zhejiang Kehua Instrument Co., Ltd	KD-P
Oven	Tianjin Laibo Rui Instrument Equipment Co., Ltd	GFL-230
Freezing microtome	Thermo	CRYOSTAR NX50
Adhesive Slides (Paraffin Sections) (White Painted)	Servicebio	G6012-1
Adhesive Slides (Frozen Sections) (White Painted)	Servicebio	G6012-2
Cover glass	Citotest Labware Manufacturing Co.,Ltd	10212432C
Upright optical microscope	Nikon	NIKON ECLIPSE E100
Imaging system	Nikon	NIKON DS-U3

1.2 Major reagents

Name	Producer	Code
Ethanol	SCRC	100092683
Xylene	SCRC	10023418
Environmental Friendly Dewaxing Transparent Liquid	Servicebio	G1128-1L
Paraformaldehyde Fixative (Neutral)	Servicebio	G1101
H&E Staining Kit	Servicebio	G1003
Neutral gum	SCRC	10004160

2. Tissue section preparation

The corresponding tissue sections were prepared according to experimental SOP of Servicebio

including pathological tissue sampling and fixation, embedding, paraffin section, frozen section, and other experiments.

3. Experimental procedure

3.1 Dewaxing and hydration: The paraffin sections were immersed in sequence in Environmental Friendly Dewaxing Transparent Liquid I for 20min - Environmental Friendly Dewaxing Transparent Liquid II for 20min - Anhydrous ethanol I for 5min - Anhydrous ethanol II for 5min - 75% Ethyl alcohol for 5min, and then rinsed with tap water.

Rewarming and fixing: The frozen sections were removed from the -20℃ refrigerator and restored to room temperature, fixed with tissue fixating solution for 15min, and then rinsed with running water.

3.2 Hematoxylin Staining： Put sections into Hematoxylin solution for 3-5 min, rinse with tap water. Then treat the section with Hematoxylin Differentiation solution, rinse with tap water. Treat the section with Hematoxylin Bluing solution, rinse with tap water.

3.3 Eosin staining: Place the sections in sequence in 85% ethanol for 5 min - 95% ethanol for 5 min- Eosin dye for 5 min.

3.4 Dehydration and sealing: Put the sections into absolute ethanol I for 5min-absolute ethanol II for 5min-absolute ethanol III for 5min-xylene I for 5min-xylene II for 5min, sealing with neutral gum.

3.5 Microscope inspection, image acquisition and analysis.

4. Interpretation of results

The nucleus is blue and the cytoplasm is red.

5. Precautions

5.1 Pay attention to the differentiation degree of nucleus;

5.2 Pay attention to the potency of hematoxylin and eosin, and change the dyeing solution in time.
