# Supplementary material for: A novel rabbit model of atherosclerotic vulnerable plaque established by cryofluid-induced endothelial injury
Source: Sci Rep. 2024 Apr 24;14:9447. doi: 10.1038/s41598-024-60287-0 (PMC11043414; doi:10.1038/s41598-024-60287-0)
Supplement: Supplementary file 5 — Supplementary Information 5. [file 41598_2024_60287_MOESM5_ESM.pdf]

Masson staining experiment report

1. Apparatus and reagents

1.1 Major apparatus

Name	Producer	Model
Dehydrator	DIAPATH	Donatello
Embedding machine	Wuhan Junjie Electronics Co., Ltd	JB-P5
Pathology slicer	Shanghai Leica Instrument Co., Ltd	RM2016
Frozen platform	Wuhan Junjie Electronics Co., Ltd	JB-L5
Tissue spreader	Zhejiang Kehua Instrument Co., Ltd	KD-P
Oven	Tianjin Laibo Rui Instrument Equipment Co., Ltd	GFL-230
Freezing microtome	Thermo	CRYOSTAR NX50
Adhesive Slides (Paraffin Sections) (White Painted)	Servicebio	G6012-1
Adhesive Slides (Frozen Sections) (White Painted)	Servicebio	G6012-2
Cover glass	Citotest Labware Manufacturing Co.,Ltd	10212432C
Upright optical microscope	Nikon	NIKON ECLIPSE E100
Imaging system	Nikon	NIKON DS-U3

1.2 Major reagents

Name	Producer	Code
Ethanol	SCRC	100092683
Xylene	SCRC	10023418
Environmental Friendly Dewaxing Transparent Liquid	Servicebio	G1128-1L
Paraformaldehyde Fixative (Neutral)	Servicebio	G1101
Masson dye solution set	Servicebio	G1006
Haematoxylin Differentiate Solution	Servicebio	G1039
Neutral gum	SCRC	10004160

2. Tissue section preparation

The corresponding tissue sections were prepared according to experimental SOP of Servicebio

including pathological tissue sampling and fixation, embedding, paraffin section, frozen section, and other experiments.

3. Experimental procedure

3.1 Dewaxing and hydration: The paraffin sections were immersed in sequence in Environmental Friendly Dewaxing Transparent Liquid I for 20min - Environmental Friendly Dewaxing Transparent Liquid II for 20min - Anhydrous ethanol I for 5min - Anhydrous ethanol II for 5min - 75% Ethyl alcohol for 5min, and then rinsed with tap water.

Rewarming and fixing: The frozen sections were removed from the -20°C refrigerator and restored to room temperature, fixed with tissue fixating solution for 15min, and then rinsed with running water.

3.2 The slices were soaked in Masson A overnight, rinse with tap water.

3.3 Masson B and Masson C were prepared into Masson solution according to the ratio of 1:1. Then stain with Masson solution for 1 min, rinse with tap water. Differentiate with 1% hydrochloric acid alcohol for several seconds, rinse with tap water.

3.4 Soak the slices in Masson D for 6 min, rinse with tap water;

3.5 Masson E for 1 min;

3.6 Do not wash the slides; slightly drain directly into Masson F for 2-30s.

3.7 Rinse the slices with 1% glacial acetic acid and then dehydration with two cup of anhydrous ethanol.

3.8 Clearing and sealing: slides were soaked in 100% ethanol for 5 min; Xylene for 5 min; finally sealed with neutral gum.

3.9 Microscope inspection, image acquisition and analysis.

4. Interpretation of results

The collagen fibers are blue; Muscle fibers, cellulose and red blood cells are red.

5. Precautions

5.1 After treat the slices with Masson A , rinse with the water should be quickly. It is sufficient to just wash the slices clean.

5.2 Replace the mixed dye solution according to the number of slices. And it can also be formulated when you need.

5.3 The staining degree of Masson D solution should be well controlled. The collagen part cannot be red, which will affect the subsequent coloration of Masson F solution;

5.4 During the differentiation of glacial acetic acid, if it is over-differentiated, the collagen blue will be too light, if it is insufficiently differentiated, it will be easily superimposed with red to become purple-blue.
