# Supplementary material for: A novel rabbit model of atherosclerotic vulnerable plaque established by cryofluid-induced endothelial injury
Source: Sci Rep. 2024 Apr 24;14:9447. doi: 10.1038/s41598-024-60287-0 (PMC11043414; doi:10.1038/s41598-024-60287-0)
Supplement: Supplementary file 6 — Supplementary Information 6. [file 41598_2024_60287_MOESM6_ESM.pdf]

Oil Red O staining experiment report

1. Apparatus and reagents

1.1 Major apparatus

Name	Producer	Model
Freezing microtome	Thermo	CRYOSTAR NX50
Microtome blades	LEICA	LEICA 819
Adhesive Slides (Frozen Sections) (White Painted)	Servicebio	G6012-2

1.2 Major reagents

Name	Producer	Code
Paraformaldehyde Fixative (Neutral)	Servicebio	G1101
Oil Red O solution	Servicebio	G1015
Hematoxylin solution	servicebio	G1004
Differentiation solution	servicebio	G1039
Bluing solution	servicebio	G1040
Glycerin gelatin mounting medium	Servicebio	G1402
Isopropanol	SCRC	80109218

2. Tissue section preparation

The corresponding tissue sections were prepared according to experimental SOP of Servicebio including pathological tissue sampling and fixation, OCT embedding, frozen section, and other experiments.

3. Experimental procedure

3.1 Frozen section fixation: Remove the frozen section from the -20℃ refrigerator and restore it to room temperature, fix it with tissue fixing solution for 15min, wash with tap water, and dry.

3.2 Oil red dyeing: 6 parts of saturated oil red O dye solution and 4 parts of distilled water were fully mixed and homogenized, left to rest at 4℃ overnight, filtered once with qualitative filter paper the next day, placed at 4℃ for 24 hours and filtered again to obtain oil red O working solution. Immerse the slices in the oil red dye solution for 8-10min (cover to avoid light).

3.3 Background differentiation: Take out the slices, stay for 3s, and then immerse in two cylinders of 60% isopropyl alcohol for differentiation, 3s and 5s respectively. The slides were immersed in 2 tanks of pure water for 10s each.

3.4 Hematoxylin staining: Take out the slides, stay for 3s, dip in hematoxylin for 3-5min and

soak in 3 tanks of pure water for 5s, 10s and 30s respectively. The differentiation solution differentiated for 2-8s, 2 tanks of distilled water washed for 10s each, and the blue solution for 1s. The slides were gently immersed in 2 tanks of tap water for 5s and 10s each, and the staining effect was checked by microscopy.

3.5 Sealing: Seal the slides with glycerin gelatin.

3.6 Microscope inspection, image acquisition and analysis.

4. Interpretation of results

The lipid droplets are orange-red to bright red, and the nucleus is blue.

5. Precautions

5.1 If it is a frozen section of fresh tissue, it needs to be fixed and then stained; if the tissue is fixed then freeze the section, the sections can be dried and dyed directly.

5.2 The whole operation process pay attention to the slow action, should not be too large, so as to avoid fat loss or displacement.

5.3 Dyeing results cannot be stored for a long time, should be observed and photographed as soon as possible after sealing.

5.4 Glycerin gelatin is solidified at room temperature, generally stored in the oven at 60°C. If there are bubbles after sealing, do not press the slide and do not forcibly pull off the cover slide. The slide can be put into warm water, let the cover slide fall off by itself, and then reseal the slides to prevent fat displacement.
